# York County Hospital: "Truth" and the Chairman of the House Committee

**Published:** 1913-04-12

**Authors:** 


### YORK COUNTY HOSPITAL.

### "Truth" and the Chairman of the House Committee.

We reproduce below a letter from the Dean of York which has appeared in the Yorkshire papers, together with *Truth's* comments thereon, published in its issue of the 2nd instant :—

### The Dean of York's Letter.

Sir,—I presided at the meeting at the hospital yesterday [Tuesday, March 18], but I am sorry to say that owing to my increasing deafness I heard very little of what was said. A sub-committee, however, was appointed, which has already met and issued a statement to the press, and in due time we shall see what comes of it.

But, in the meanwhile, I am anxious to lift up my voice in behalf of the hospital staff, who, I know, feel very keenly the virulent imputations which are being circulated about them. Without claiming for them, or for any body of workers, absolute perfection, I am convinced that there is no reason for at once flinging away any confidence in them. But as my testimony would certainly be considered partial and biassed, I wish to invoke "The Working Men's Hospital Fund Society" to give us their experience of the treatment of patients at the hospital. They represent a very large body, not only of subscribers, but also of benefit members who have received personal treatment at the hospital, both as in and out patients, and I would ask them whether, as their annual subscriptions are not only maintained, but increased, that is not the best practical test of the efficiency of the institution. York men do not, as a rule, subscribe for what they find to be not worth having—for careless attention, bad accommodation, and insufficient treatment. And we have three of the members who are also members of the Hospital Committee, and I have never heard any word of complaint from them. This is, after all, the best test that we can have. And if that is the case, we may condone the Suffragette action on the part of two tyros, even though they suffered from "lack of court etiquette," from "the matron wishing to argue the matter," and the unvaried menu of their evening dinner.

The public temper has been very much excited by the mortuary incident as of something unpardonable. No doubt it is much to be regretted by all, and I am surprised that, with the callous quotation of the matron's words, there is not also mention of her agitation and the distress of herself and the nurses when they found what had inadvertently happened. But such an episode is by no means an isolated experience. I doubt if there is a single medical man of any long professional practice who has not had, in some way, similar experience. I am only a layman in these matters, but I have known of one at least.

I therefore desire to lift up my voice for fair play for the hospital staff. If they can be proved to be a band of persistent and subtle evildoers to the sick and suffering, *cadit quæstio*; but until that is established I would express my conviction, from the experience of the last thirty years, that the sick and suffering inmates, as well as the subscribers, have been, and are still, most fortunate in securing a succession of kind, attentive, and unflagging labourers in a very difficult and exhausting work, and I cordially endorse the very sensible and practical article which the *Yorkshire Herald* published last Saturday on this matter.—I remain, yours faithfully,

Arthur P. Purey-Cust,
Chairman of House Committee,
York Hospital.

Deanery, York,

March 19, 1913.

### "Truth" on the Dean's Letter.

"*Apropos* of the York Hospital scandal, a remarkable letter from the Dean of York, in defence of the staff of the York County Hospital, appeared in the *Yorkshire Herald*. The Dean is chairman of the House Committee of the hospital, and, pending the holding of the promised inquiry, one would have thought that he would have been well advised to have awaited the result before rushing into print. He seems to have lost sight of the fact that the House Committee is just as much on its trial as the staff for whom he takes up the cudgels. A committee under whose management such a state of things as that described by the late resident medical officers of the institution could be allowed to exist would show itself hopelessly incompetent.

"In his apologia the Dean remarks that "the public temper has been very much excited by the mortuary incident as of something unpardonable," and he goes on to say that such an episode is by no means an isolated experience. This may be the Dean's experience at York, but such a belief argues but a very small acquaintance with modern hospital methods or modern hospital management. The majority of hospital committees would, I am sure, consider that the transference of a patient while alive to the mortuary was "something unpardonable," and would at once have taken drastic steps to prevent the recurrence of such an "incident" without the spur of public opinion. However, as the Dean is an exceedingly elderly man—he commences his letter by stating that owing to deafness he heard very little of what passed at the committee meeting which dealt with the allegations—the only deduction to be drawn from his letter is that the House Committee should be reconstituted of younger men in full possession of their faculties, and with more modern experience to guide them."

---

Further correspondence on this subject will be found in "The Editor's Letter Box" on page 53 of the present issue.—Ed. The Hospital.

